# ECDC Director vacancy now open

**DOI:** 10.2807/1560-7917.ES.2023.28.22.2306011

**Published:** 2023-06-01

**Authors:** Eurosurveillance editorial team

**Affiliations:** 1 European Centre for Disease Prevention and Control (ECDC), Stockholm, Sweden

The recruitment process for the position of ECDC Director is handled by the European Commission.

 

The deadline for the submission of applications is 26 June 2023, 12:00 noon CEST.

For detailed information on the job description, requirements and the application procedure, please visit:

https://eur-lex.europa.eu/legal-content/EN/TXT/?uri=OJ%3AJOC_2023_185_A_0001

